# Unveiling the role of miR-137-3p/miR-296-5p/SERPINA3 signaling in colorectal cancer progression: integrative analysis of gene expression profiles and in vitro studies

**DOI:** 10.1186/s12920-023-01763-w

**Published:** 2023-12-12

**Authors:** Huimin Liu, Xingxing Wu, Dandan Wang, Quanxi Li, Xin Zhang, Liang Xu

**Affiliations:** 1 Department of General Surgery, The Second People’s Hospital of Lianyungang, Lianyungang, China; 2 Department of Pediatric Surgery, The Second People’s Hospital of Lianyungang, Lianyungang, China

**Keywords:** Colorectal cancer, Metastasis, Bioinformatics analysis, DEGs, SERPINA3, miR-137-3p, miR-296-5p

## Abstract

**Background:**

Colorectal cancer (CRC) is a prevalent malignancy worldwide, with increasing incidence and mortality rates. Although treatment options have improved, CRC remains a leading cause of death due to metastasis. Early intervention can significantly improve patient outcomes, making it crucial to understand the molecular mechanisms underlying CRC metastasis. In this study, we performed bioinformatics analysis to identify potential genes associated with CRC metastasis.

**Methods:**

We downloaded and integrated gene expression datasets (GSE89393, GSE100243, and GSE144259) from GEO database. Differential expression analysis was conducted, followed by Gene Ontology (GO) functional enrichment analysis and Kyoto Encyclopedia of Genes and Genomes (KEGG) pathway enrichment analysis. The hub gene SERPINA3 was selected for further in vitro functional studies. Additionally, the role of miR-137-3p/miR-296-5p/ Serpin family A member 3 (SERPINA3) in CRC cell function was investigated using in vitro assays.

**Results:**

Analysis of the gene expression datasets revealed differentially expressed genes (DEGs) associated with CRC metastasis. GO analysis showed enrichment in biological processes such as blood coagulation regulation and wound healing. Cellular component analysis highlighted extracellular matrix components and secretory granules. Molecular function analysis identified activities such as serine-type endopeptidase inhibition and lipoprotein receptor binding. KEGG analysis revealed involvement in pathways related to complement and coagulation cascades, cholesterol metabolism, and immune responses. The common DEGs among the datasets were further investigated. We identified SERPINA3 as a hub gene associated with CRC metastasis. SERPINA3 exerted enhanced effects on migration, proliferation and epithelial-mesenchymal transition (EMT) and inhibitory effects on caspase-3/-9 activities in HT29 and SW620 cells. MiR-137-3p overexpression increased activities of caspase-3/-9, decreased migration and proliferation, and also repressed EMT in HT29 cells, which were obviously attenuated by SERPINA3 enforced overexpression. Consistently, SERPINA3 enforced overexpression also largely reversed miR-296-5p mimics-induced increased in activities of caspase-3/-9, decrease in migration, proliferation and EMT in HT29 cells.

**Conclusion:**

Through bioinformatics analysis, we identified potential genes associated with CRC metastasis. The functional studies focusing on SERPINA3/miR-137-3p/miR-296-5p further consolidated its role in regulating CRC progression. Our findings provide insights into novel mechanisms underlying CRC metastasis and might contribute to the development of effective treatment strategies. However, the role of SERPINA3/miR-137-3p/miR-296-5p signaling in CRC still requires further investigation.

**Supplementary Information:**

The online version contains supplementary material available at 10.1186/s12920-023-01763-w.

## Introduction

Colorectal cancer (CRC) is one of the most common malignant tumors worldwide [1]. In 2023, the American Society of Clinical Oncology predicts that the incidence and mortality of colorectal cancer will rank third among all malignant tumors [2]. With advancement in treatment methods, the survival rate of colorectal cancer patients has significantly increased, leading to an improved quality of life. However, in recent years, as economic levels have risen and lifestyle and dietary habits have changed, the incidence of colorectal cancer has been on the rise [2–4]. It is characterized by an earlier age of onset, lower staging, and higher malignancy [3–5]. Currently, surgical resection is the most common and effective treatment method for colorectal cancer. Radiation therapy, chemotherapy, immunotherapy, and targeted therapy can to some extent, extend the survival rate of patients and enhance their quality of life, but the mortality rate remains high [6, 7]. When diagnosed with colorectal cancer, approximately 80% of patients already have experienced localized disease, while 20% have distant metastases [8, 9]. The most common sites of metastasis include the peritoneum, liver, regional lymph nodes, and lungs. Metastasis is the primary cause of death in CRC patients. Early intervention treatment in the early stages of colorectal cancer can result in five-year survival rate for patients exceeding 90% [10, 11]. Therefore, understanding the specific molecular mechanisms of colorectal cancer metastasis holds significant implications for developing effective treatment strategies in clinical practice.

Bioinformatics is an emerging interdisciplinary field that involves the acquisition, processing, storage, dissemination, analysis, interpretation, and application of biological information using computers [12]. It aims to unravel the mysteries behind a vast amount of complex biological data. Currently, bioinformatics analyses, such as gene expression profiling, metabolic network analysis, and proteomics data analysis are being widely conducted globally [13–17]. These analyses have gradually become important emerging research areas within the field of bioinformatics. In recent years, numerous genes related to tumor progression have been identified by researchers through database analysis methods [13–17]. These genes could potentially serve as tumor markers in the future. For example, Zhou et al. performed bioinformatics analysis and revealed that DEAD-box helicase 10 promotes CRC cell metastasis through the splicing of ribosomal protein L35 [18]. A study analyzed the expression of serine and arginine rich splicing factor 6 (SRSF6) in 311 CRC samples from The Cancer Genome Atlas and Gene Expression Omnibus (GEO) database, and demonstrated that SRSF6-regulates alternative splicing to promote CRC metastasis [19]. Wang et al. conducted an analysis on GSE49355 and GSE81582, identifying inositol monophosphatase 2 as the hub gene associated with colorectal cancer and liver metastasis [20].

Serpin family A member 3 (SERPINA3), also known as α-1-antichymotrypsin, belongs to the serpin family and serves as an acute phase protein that exhibits an increase in blood levels during inflammation [21, 22]. SerpinA3’s inhibitory effect is notably directed toward neutrophil cathepsin G, mast cell chymases, and pancreatic chymotrypsin [21]. The primary sources of serpinA3 synthesis are hepatocytes, bronchial epithelial cells, and monocytes, with additional expression found in various organs, including the kidney, brain, and prostate [21]. SERPINA3 appears to exhibit cancer- and location-specific biological roles, functioning as either a facilitator or inhibitor of tumor growth in various types of cancer [23]. Elevated levels of proteinase inhibitors, such as serpinA3, have been associated with a poor prognosis and increased malignancy in gastric cancer [24]. Dimberg et al. suggested that an altered S concentration in CRC tissue may be a potential biomarker in CRC progression [25]. However, the mechanism of action and the effectors of SERPINA3 in cancer remain obscure.

In this study, we explored potential genes associated with CRC metastasis through bioinformatics analysis. Firstly, we downloaded the datasets GSE89393, GSE100243 and GSE144259 from the GEO database, which contain gene information from CRC patients. We then performed preliminary processing and integration of these datasets to identify differentially expressed genes (DEGs). Furthermore, we conducted GO (Gene Ontology) functional enrichment and KEGG (Kyoto Encyclopedia of Genes and Genomes) pathway [26] analysis on the target genes to explore their potential functions. Additionally, we selected SERPINA3 for further in vitro functional studies. Finally, we determined the role of miR-137-3p/miR-296-5p/SERPINA3 in CRC cell function through in vitro assays.

## Materials and methods

### Metadata for GEO datasets

GEO datasets analyzed in the study include GSE89393, GSE100243 and GSE144259. The GSE89393 dataset contains three groups: normal colon = 6 samples, colon tumor = 5 samples, liver metastasis = 6 samples; GSE100243 contains three groups: normal colon = 6 samples, primary colon tumor = 8 samples, lymph node metastasis = 8 samples; GSE144259 contains normal colon = 3 samples, colon tumor = 3 samples, liver metastasis = 3 samples. This study retrieved DEGs between cancerous group and metastasis group.

### GEO datasets process

DEGs between cancerous and metastasis groups from GSE89393, GSE100243 and GSE144259 CRC patients were analyzed by online GREIN-ILINCS tool. Selection criteria for DEGs were based on following: |log(fold change)|> 1.2, adjusted *p* values < 0.05. The extracted DEGs were saved in excel files for further analysis. Heatmap illustrating DEGs between cancerous and metastasis groups was generated by using GREIN-ILINCS tool. Common DEGs among GSE89393, GSE100243 and GSE144259 were plotted using Veen diagram.

### Enrichment analysis

DEGs from GSE89393, GSE100243 and GSE144259 were subjected to GO and KEGG enrichment analysis [26] by using EnrichR tool. The GO enrichment includes “GO_biological process”, “GO_cellular component” and “GO_molecular function”.

### Protein-protein interaction (PPI) network construction

Common DEGs were extracted for PPI network construction using STRING tool. The constructed network was illustrated by Cytoscape software. The criteria for constructing the PPI network was set as follows: meaning of network edges = evidence, confidence = 0.7, network type = full STRING network.

### Survival analysis

Survival analysis illustrating association between common DEGs and survival status (OS = “overall survival”; RFS= “relapse-free survival”; PPS= “post-progression survival”) was carried out using KM plotter. In OS analysis cohort, 551 CRC patients were included; in RFS analysis cohort, 145 patients were included; in PPS analysis cohort, 1342 CRC patients were included.

### Expression analysis of common DEGs

Gene Expression Profiling Interactive Analysis (GEPIA) database [27] was utilized to analysis the expression of common DEGs, and *P* < 0.05 was statistically significant. The database includes 41 normal colon tissues and 275 cancerous colon tissues.

### Expression of SEPRINA3 in colon Cancer tissues with different stages and in immune cells

The expression of SEPRINA3 in colon cancer tissues with different stages was analyzed by using GEPIA2 [28]. The expression of SEPRINA3 in various immune cells was analyzed by CIBERSORT through the GEPIA2021 [29].

### Cell lines and culture

The HT29 (#HTB-38) and SW620 (#CCL227) cell lines derived from CRC were acquired from ATCC (Manassas, USA). HT29 and SW620 cells were maintained in DMEM medium (#12,491,015) supplemented with 10% fetal bovine serum (#A5256701; FBS, Thermo Fisher Scientific, Waltham, USA) in a humidified incubator at 37 °C with 5% CO_2_.

### SiRNAs, miRNAs, plasmids and cell transfections

SiRNAs specifically targeting SERPINA3 (referred to as si-SERPINA3#1 and si-SERPINA3#2) were synthesized by Ribobio (Guangzhou, China). A scrambled siRNA (si-NC) was used as the negative control. Ribobio (Guangzhou) also provided the miR-137-3p mimics, miR-296-5p mimics, and their respective control molecules. The pcDNA vector and SERPINA3-overexpressing vector (pcDNA-SERPINA3) were obtained from Ribobio (Guangzhou). For cell transfections, siRNAs, miRNAs, and plasmids were introduced into HT29 or SW620 cells using Lipofectamine 2000 reagent (Invitrogen, Carlsbad, USA) following the manufacturer’s instructions.

### Quantitative real-time PCR

For RNA extraction from HT29 and SW620 cells, TRIzol reagent (#15,596,026; Invitrogen) was used. The extracted RNA was then converted into cDNA using the High Capacity cDNA Reverse Transcription Kit (#4,368,814; Thermo Fisher Scientific). Real-time PCR was carried out on an ABI7900 system (Applied Biosystems) using SYBR Green PCR Master Mix (#4,309,155; Thermo Fisher Scientific). MiRNA expression was normalized using U6, while mRNA expression was normalized using GAPDH. The relative expression of the respective targets was calculated using the 2^−ΔΔCt^ method.

### Caspase-3/-9 activity

The activities of caspase-3 and caspase-9 were determined using the caspase-3 (#C1116) and caspase-9 (#C1158) activity kits (Beyotime) respectively. In brief, caspase-3 hydrolyzes Ac-DEVD-pNA, while caspase-9 cleaves labeled substrates LEHD-pNA. The concentration of the released chromophore pNA was measured at 405 nm using a spectrophotometer. The pNA concentration was determined using a calibration curve prepared with pNA standards to quantify respective activities.

### Wound healing assay

To evaluate migratory capacity of SW620 and HT29 cells, a wound-healing assay was performed. The cell monolayer was wounded using 200 μl pipette tips after different treatments, and images of the same site on the monolayer were captured at 0 and 48 h using a microscope (Zeiss). The gap size was measured using ImageJ software, and the percentage migration was calculated based on the wound size at 0 h.

### Cell counting Kit-8 (CCK-8) assay

To assess relative cell proliferation, HT-29 and SW620 cells with different treatments were seeded into 96-well microplates at a density of 5000 cells. CCK-8 assay (#C0039; Beyotime) was then performed following the manufacturer’s instructions. Briefly, 10 μL of CCK-8 working solution was added per 100 μL of medium in the microplates, and the cells were incubated for 2 h. The OD_450_ value was measured using a microplate reader (Bio-Tek).

### Luciferase assay

The binding sites between miR-137-3p/miR-296-5p and SERPINA3 3’ untranslated region (3’UTR) were predicted by miRDB [30]. The corresponding wild type and mutant SEPRIN3 3’UTR were subcloned into pGL3 vector (#E1751; Promega). For luciferase assay, HT29 cells were co-transfected with respective miRNAs and constructed reporter vector. At 48 h after transfection, the luciferase activity was determined by the Dual-Luciferase Reporter assay system (#E1910; Promega).

### Statistical analysis

The data are presented as means ± standard deviation. For comparisons, appropriate statistical tests including the Student’s t-test and one-way analysis of variance followed by Bonferroni’s post-hoc tests were performed. All analyses were conducted using GraphPad Prism software (Version 6.0, GraphPad Software). Differences were considered significant at *P* < 0.05.

## Results

### Analysis of DEGs from GSE89393, GSE100243 and GSE144259

**Fig. 1:**
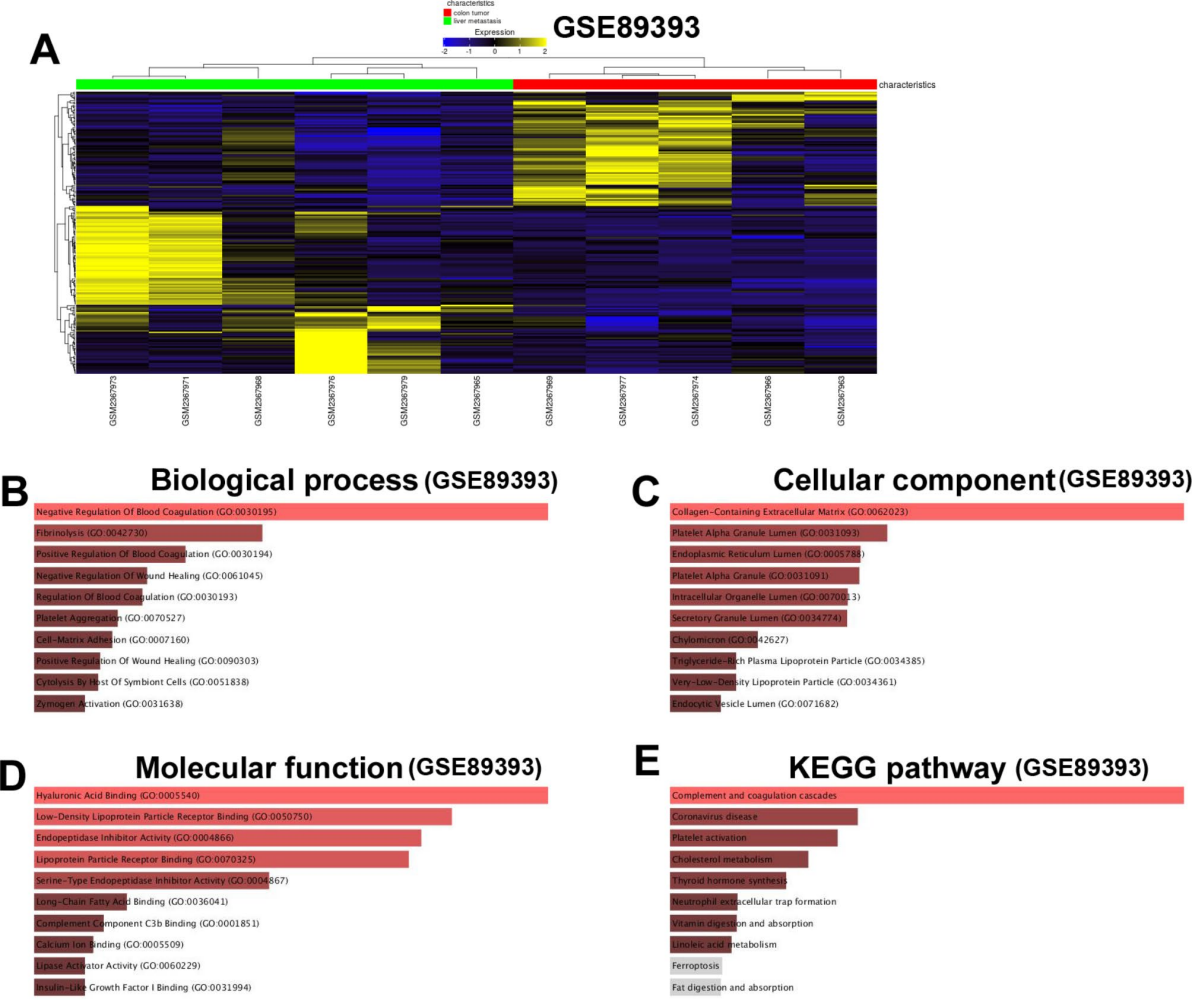
Enrichment analysis of DEGs from GSE89393. (**A**) Heatmap illustrates the DEGs from GSE100243 between cancerous and metastatic group. (**B**) “GO_biological process” of DEGs. The horizontal axis represents the number of genes, and the y-axis represents biological process. (**C**) “GO_cellular component” enrichment of DEGs. The horizontal axis represents the number of genes, and the y-axis represents cellular component. (**D**) “GO_molecular function” of DEGs. The horizontal axis represents the number of genes, and the y-axis represents molecular function. (**E**) “KEGG pathway” of DEGs. The horizontal axis represents the number of genes, and the y-axis represents KEGG pathway names

Firstly, we extracted corresponding DEGs from GSE89393, GSE100243 and GSE144259, and these DEGs were compared between cancerous tissues and metastatic tissues in patients with colon cancer. In GSE89393, 33 DEGs were extracted (Fig. 1A). GO_biological process analysis revealed that DEGs were enriched in “Negative Regulation Of Blood Coagulation”, “Fibrinolysis”, “Positive Regulation Of Blood Coagulation” and so on (Fig. 1B). GO_cellular component analysis revealed that DEGs were enriched in “Collagen-Containing Extracellular Matrix”, “Platelet Alpha Granule Lumen”, “Endoplasmic Reticulum Lumen”, and so on (Fig. 1C). GO_molecular function analysis revealed that DEGs were enriched in “Hyaluronic Acid Binding”, “Low-Density Lipoprotein Particle Receptor Binding”, “Endopeptidase Inhibitor Activity” and so on (Fig. 1D). KEGG analysis revealed that DEGs were enriched in “Complement and coagulation cascades”, “Coronavirus disease”, “Platelet activation” and so on (Fig. 1E).

**Fig. 2:**
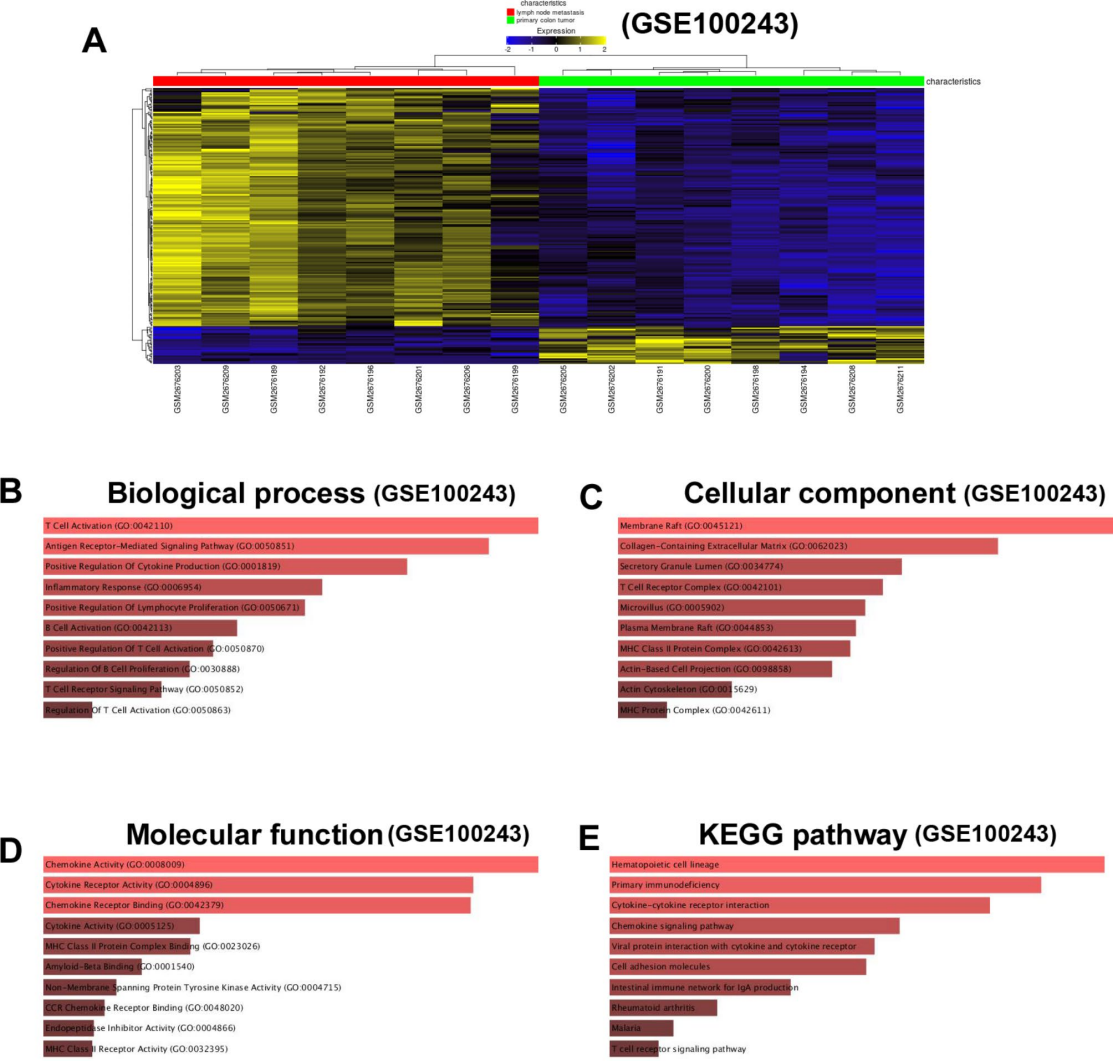
Enrichment analysis of DEGs from GSE100243. (**A**) Heatmap illustrates the DEGs from GSE100243 between cancerous and metastatic group. (**B**) “GO_biological process” of DEGs. The horizontal axis represents the number of genes, and the y-axis represents biological process. (**C**) “GO_cellular component” enrichment of DEGs. The horizontal axis represents the number of genes, and the y-axis represents cellular component. (**D**) “GO_molecular function” of DEGs. The horizontal axis represents the number of genes, and the y-axis represents molecular function. (**E**) “KEGG pathway” of DEGs. The horizontal axis represents the number of genes, and the y-axis represents KEGG pathway names

In GSE100243, 1323 DEGs were extracted (Fig. 2A). GO_biological process analysis revealed that DEGs were enriched in “T Cell Activation”, “Antigen Receptor-Mediated Signaling Pathway”, “Positive Regulation Of Cytokine Production” and so on (Fig. 2B). GO_cellular component analysis revealed that DEGs were enriched in “Membrane Raft”, “Collagen-Containing Extracellular Matrix”, “Secretory Granule Lumen” and so on (Fig. 2C). GO_molecular function analysis revealed that DEGs were enriched in “Chemokine Activity”, “Cytokine Receptor Activity”, “Chemokine Receptor Binding” and so on (Fig. 2D). KEGG analysis revealed that DEGs were enriched in “Hematopoietic cell lineage”, “Primary immunodeficiency”, “Cytokine-cytokine receptor interaction” and so on (Fig. 2E).

**Fig. 3:**
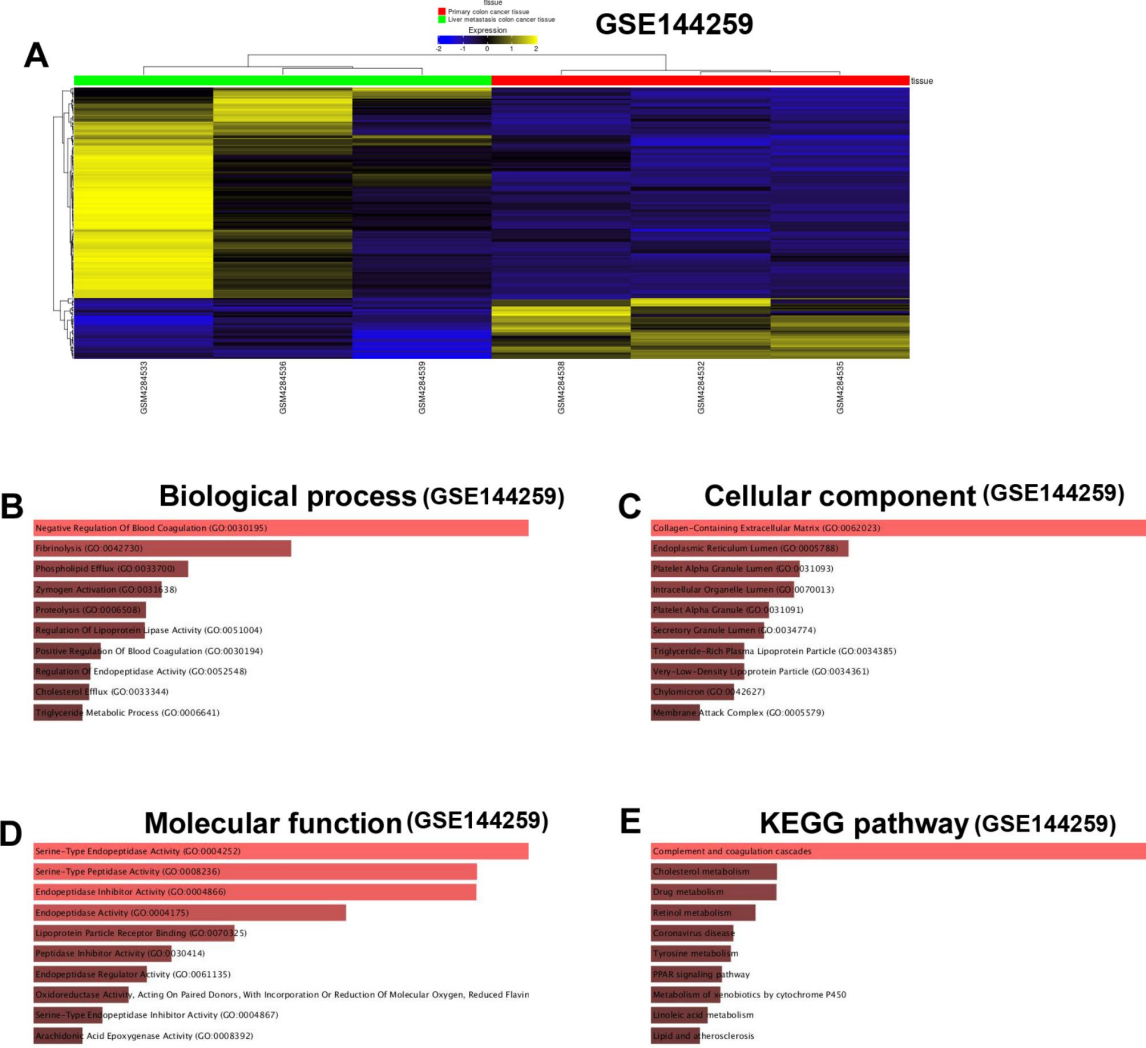
Enrichment analysis of DEGs from GSE144259. (**A**) Heatmap illustrates the DEGs from GSE144259 between cancerous and metastatic group. (**B**) “GO_biological process” of DEGs. The horizontal axis represents the number of genes, and the y-axis represents biological process. (**C**) “GO_cellular component” enrichment of DEGs. The horizontal axis represents the number of genes, and the y-axis represents cellular component. (**D**) “GO_molecular function” of DEGs. The horizontal axis represents the number of genes, and the y-axis represents molecular function. (**E**) “KEGG pathway” of DEGs. The horizontal axis represents the number of genes, and the y-axis represents KEGG pathway names

In GSE144259, 131 DEGs were extracted (Fig. 3A). GO_biological process analysis revealed that DEGs were enriched in “Negative Regulation Of Blood Coagulation”, “Fibrinolysis”, “Phospholipid Efflux” and so on (Fig. 3B). GO_cellular component analysis revealed that DEGs were enriched in “Collagen-Containing Extracellular Matrix”, “Endoplasmic Reticulum Lumen”, “Platelet Alpha Granule Lumen” and so on (Fig. 3C). GO_molecular function analysis revealed that DEGs were enriched in “Serine-Type Endopeptidase Activity”, “Serine-Type Peptidase Activity”, “Endopeptidase Inhibitor Activity” and so on (Fig. 3D). KEGG analysis revealed that DEGs were enriched in “Complement and coagulation cascades”, “Cholesterol metabolism”, “Drug metabolism” and so on (Fig. 3E).

**Fig. 4:**
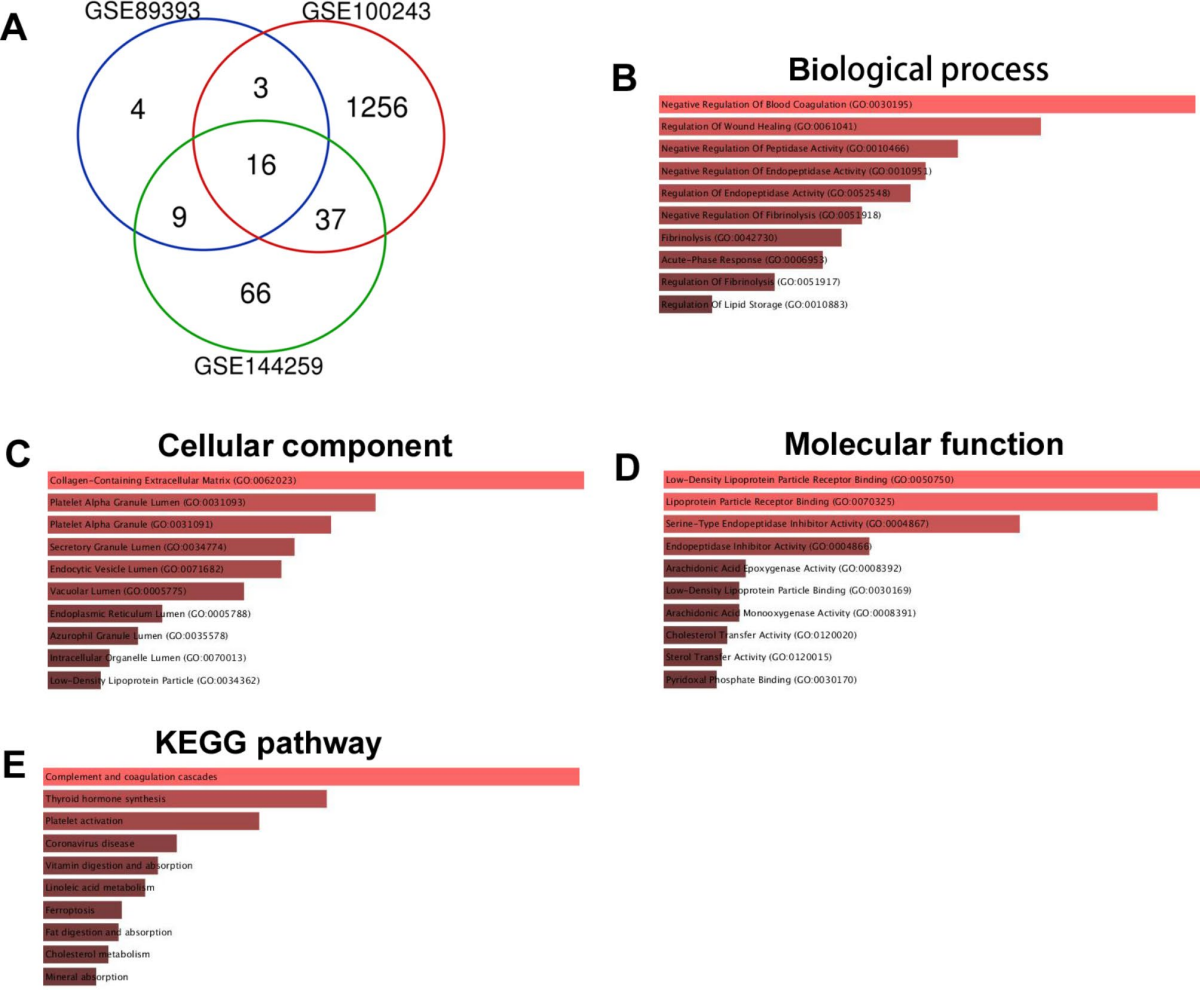
Enrichment analysis of common DEGs among GSE89393, GSE100243 and GSE144259 (**A**) Venn diagram shows the common DEGs among GSE89393, GSE100243 and GSE144259 between cancerous and metastatic group. (**B**) “GO_biological process” of common DEGs. The horizontal axis represents the number of genes, and the y-axis represents biological process. (**C**) “GO_cellular component” of common DEGs. The horizontal axis represents the number of genes, and the y-axis represents cellular component. (**D**) “GO_molecular function” of common DEGs. The horizontal axis represents the number of genes, and the y-axis represents molecular function. (**E**) “KEGG pathway” of common DEGs. The horizontal axis represents the number of genes, and the y-axis represents KEGG pathway names

Sixteen common DEGs were extracted among GSE89393, GSE100243 and GSE144259 (Fig. 4A). GO_biological process analysis revealed that DEGs were enriched in “Negative Regulation Of Blood Coagulation”, “Regulation Of Wound Healing”, “Negative Regulation of Peptidase Activity” and so on (Fig. 4B). GO_cellular component analysis revealed that DEGs were enriched in “Collagen-Containing Extracellular Matrix”, “Platelet Alpha Granule Lumen”, “Platelet Alpha Granule” and so on (Fig. 4C). GO_molecular function analysis revealed that DEGs were enriched in “Low-Density Lipoprotein Particle Receptor Binding”, “Lipoprotein Particle Receptor Binding”, “Serine-Type Endopeptidase Inhibitor Activity” and so on (Fig. 4D). KEGG analysis revealed that DEGs were enriched in “Complement and coagulation cascades”, “Thyroid hormone synthesis”, “Platelet activation” and so on (Fig. 4E).

### PPI network analysis of common DEGs

**Fig. 5:**
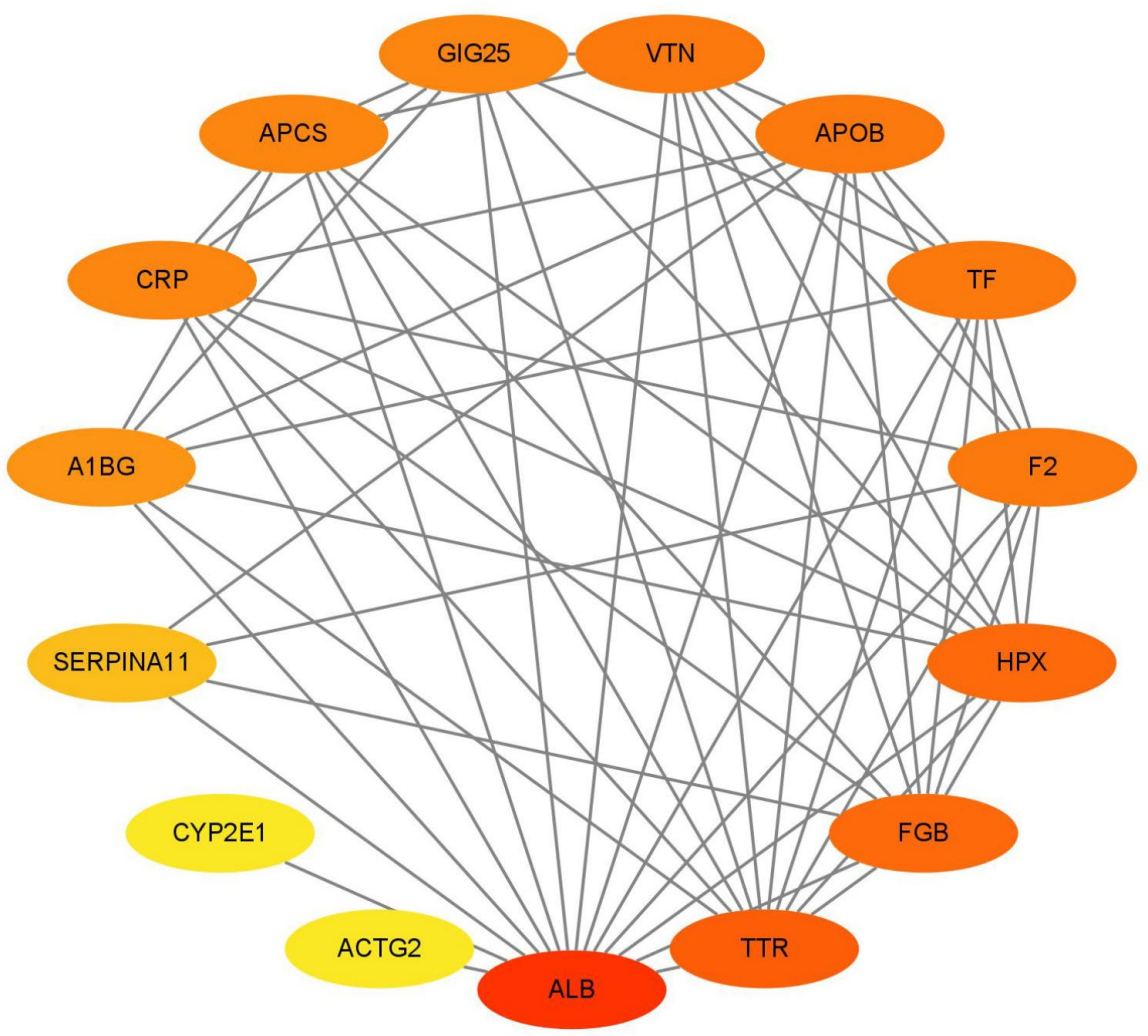
PPI network analysis of common DEGs. The network includes 15 nodes and 59 edges. Circles represent genes, lines represent the interaction of proteins between genes. Red color represents highest degree, and orange color represents intermedia degree, and yellow color represents lowest degree

Common DEGs were subjected to query using STRING tool, and the constructed PPI network was visualized by Cytoscape software. The constructed PPI network consisted of 15 nodes and 59 edges (Fig. 5). As shown in Fig. 5, circles represent genes, lines represent the interaction of proteins between genes. Red color represents highest degree, and orange color represents intermedia degree, and yellow color represents lowest degree. Albumin (ALB) has the highest degree of connectivity, while cytochrome P450 2E1(CYP2E1) has the lowest degree of connectivity (Fig. 5).

### Association between OS, PPS and RFS of CRC patients and common DEGs

**Fig. 6:**
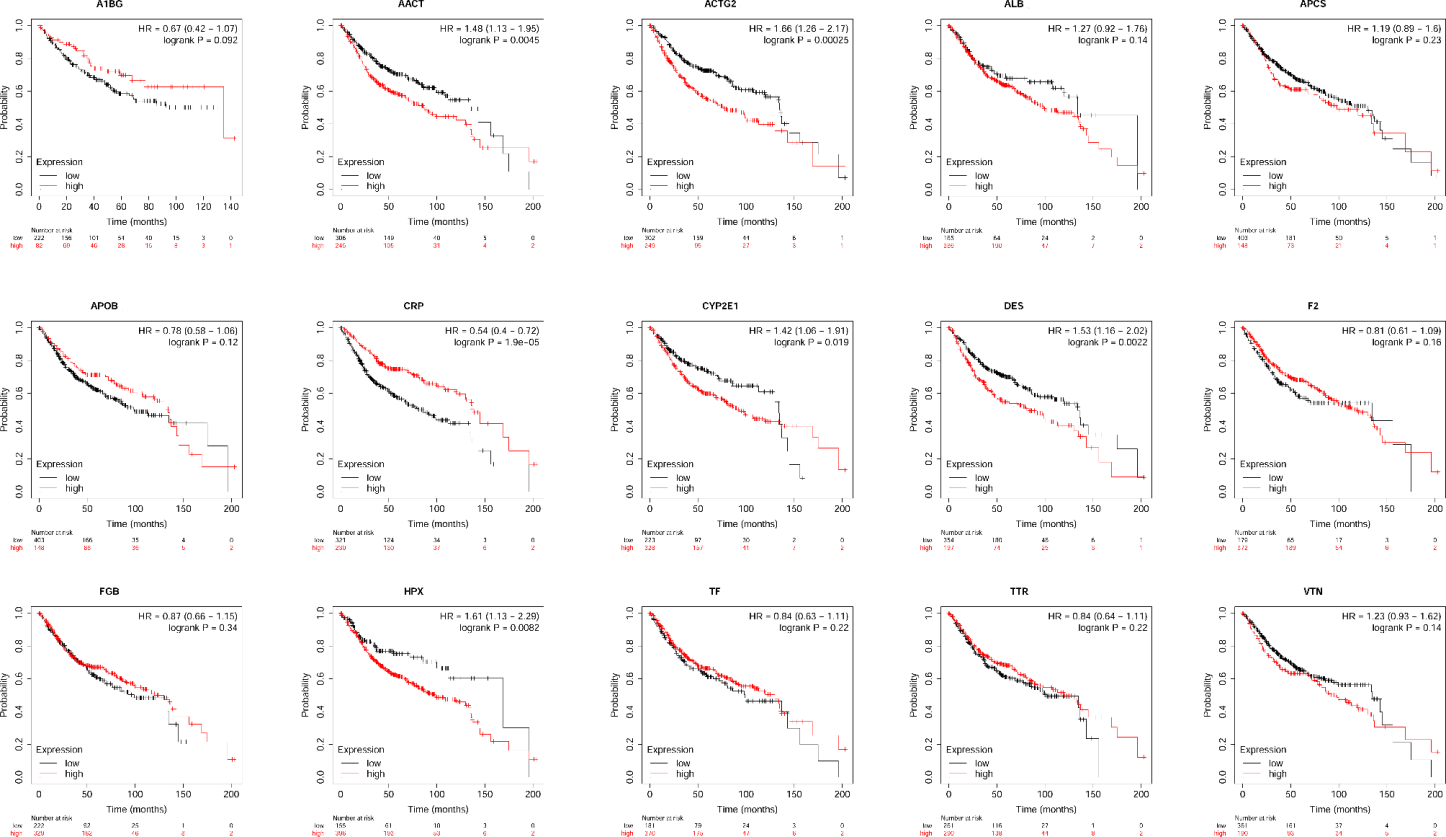
The association between OS of patients with colon cancer and common DEGs. Kaplan–Meier plot of overall survival in subjects with low versus high for genes including A1BG, AACT, ACTG2, ALB, APCS, APOB, CRP, CYP2E1, DES, F2, FGB, HPX, TF, TTR and VTN

The association between survival status and individual common DEG expression was determined by KM-Plotter too. High expression of AACT (SERPINA3), actin gamma 2 (ACTG2), CYP2E1, desmin (DES), hemopexin (HPX) was associated with poorer OS of patients (Fig. 6); while high expression of C-reactive protein (CRP) was associated with better OS of patients (Fig. 6). Additionally, alpha-1-B glycoprotein (A1BG), ALB, amyloid P component, serum (APCS), apolipoprotein B (APOB), coagulation factor II, thrombin (F2), fibrinogen beta chain (FGB), transferrin (TF), transthyretin (TTR) and vitronectin (VTN) were not associated with OS (Fig. 6).

In terms of PPS, high expression of AACT (SERPINA3), ALB, APCS, CYP2E1, TTR and VTN was associated with poorer PPS of patients (Figure S1); while high expression of F2 and HPX was associated with better PPS of patients (Figure S1). Additionally, A1BG, ACTG2, APOB, CRP, DES, FGB and TF were not associated with OS (Figure S1).

In terms of RFS, high expression of A1BG, AACT (SERPINA3), ACTG2, ALB, APCS, CRP, CYP2E1, DES, HPX, TF and VTN was associated with poorer RFS of patients (Supplementary Figure S2); while high expression of TTR was associated with better PPS of patients (Supplementary Figure S2). Additionally, ALB, APOB, F2, FGB and TF were not associated with OS (Supplementary Figure S2).

The expression of the common DEGs between normal and colon cancer tissues was analyzed using GEPIA tool. As shown in Supplementary Figure S3, ACTG2 and DES were down-regulated in cancerous tissues when compared to normal controls; while no significant differences in the expression of other genes between cancerous and normal tissues were detected (Supplementary Figure S3).

Based on the literature research, the high connectivity of SERPINA3 in the PPI network, and the prognostic role of SERPINA3 in CRC patients, SERPINA3 was selected for the subsequent analysis and in vitro studies. As shown in supplementary Figure S4, the stage plot by using GEPIA2 demonstrated the high expression of SPERINA3 in colon cancer when compared to stage II-III. We also investigated SERPINA3 expression levels in various immune cells by CIBERSORT through GEPIA2021, the results revealed that SEPRINA3 was highly expressed in the M2 macrophages (Supplementary Figure S5).

### Effects of SERPINA3 knockdown on CRC cell progression

**Fig. 7:**
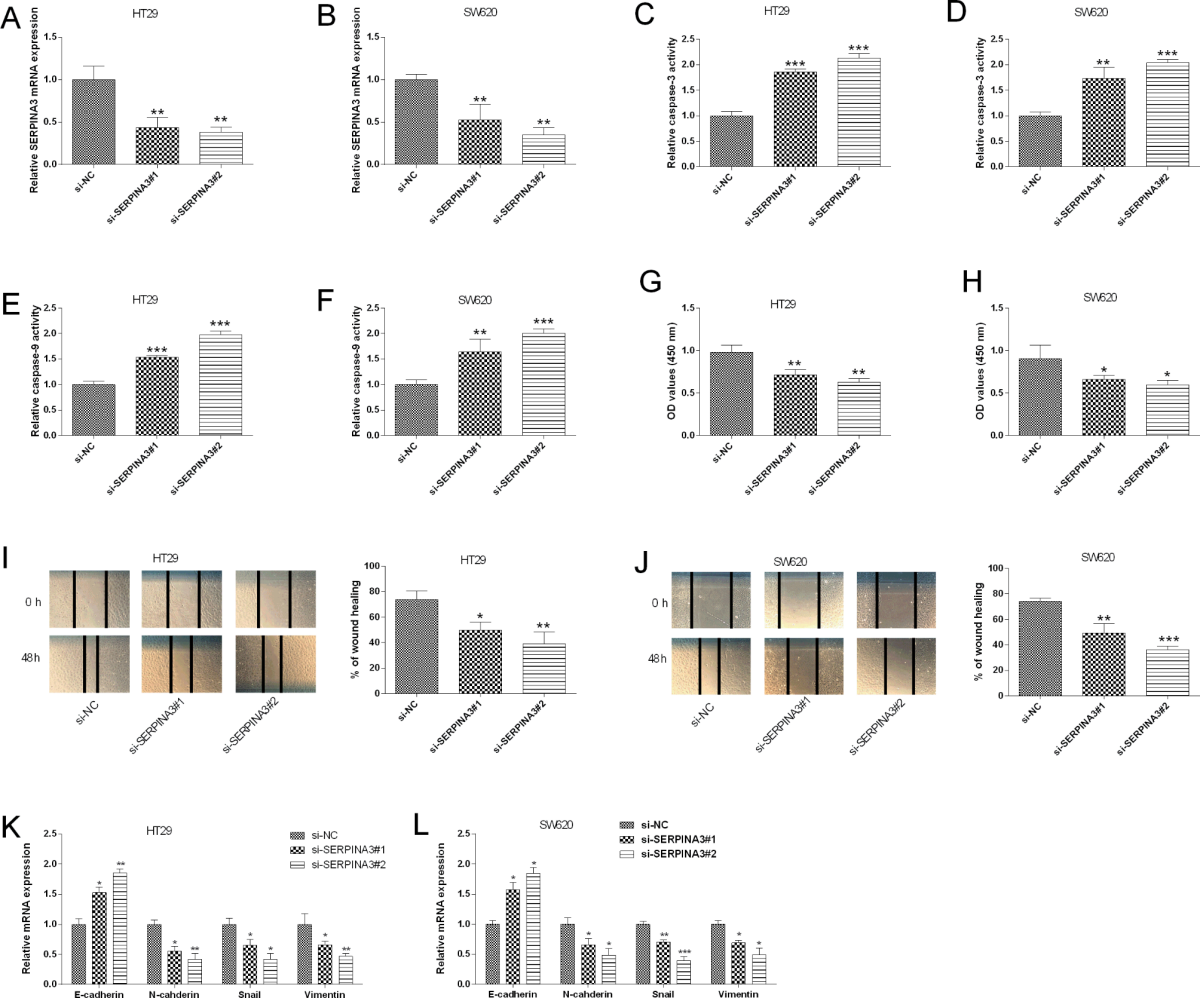
Effects of SERPINA3 knockdown on the colon cancer cell migration, proliferation and EMT. (**A**) HT29 and (**B**) SW620 cells were transfected with siRNAs targeting SERPINA3 or control siRNA, SERPINA3 expression was determined by evaluated by qRT-PCR. (**C**) HT29 and (**D**) SW620 cell caspase-3 activity was determined in different groups (si-NC, si- SERPINA3#1, si- SERPINA3#3). (**E**) HT29 and (**F**) SW620 cell caspase-9 activity was determined in different groups (si-NC, si- SERPINA3#1, si- SERPINA3#3). (**G**) HT29 and (**H**) SW620 cell proliferation was determined in different groups (si-NC, si- SERPINA3#1, si- SERPINA3#3). (**I**) HT29 and (**J**) SW620 cell migratory capacity was determined in different groups (si-NC, si- SERPINA3#1, si- SERPINA3#3). (**K**) HT29 and (**L**) SW620 cell EMT-related marker expression was evaluated in different groups (si-NC, si- SERPINA3#1, si- SERPINA3#3). N = 3. *P < 0.05, **P < 0.01 and ***P < 0.001

SERPINA3 silencing effects in two colon cancer cell lines were investigated. Transfecting siRNAs targeting SERPINA3 into HT29 and SW620 cells repressed SERPINA3 expression compared to si-NC group (Fig. 7A and B). SERPINA3 silencing potentiated caspase-3/-9 activities in HT29 and SW620 cells compared to si-NC group (Fig. 7C and F). Consistently, SERPINA3 silencing attenuated HT29 and SW620 cell proliferation (Fig. 7G and H). Additionally, transfecting siRNAs targeting SERPINA3 into HT29 and SW620 also impaired migratory ability of these cells compared to si-NC group (Fig. 7I and J). Epithelial-mesenchymal transition (EMT)-related markers were also examined in different groups, and SERPINA3 silencing up-regulated E-cadherin, but down-regulated N-cadherin, Snail and Vimentin in CRC cells (Fig. 7K and L).

### SERPINA3 was negatively regulated by mir-137-3p and miR-296-5p

**Fig. 8:**
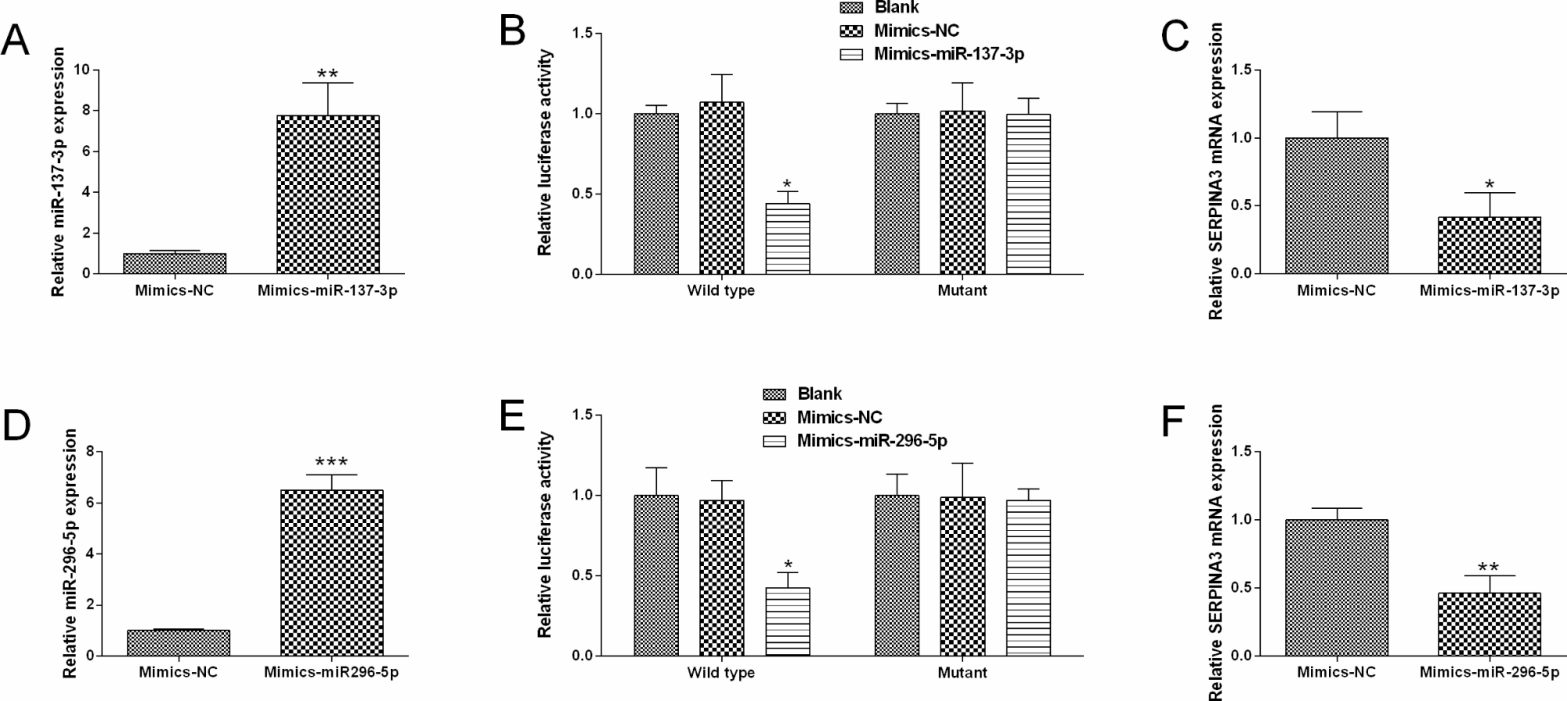
SERPINA3 was negatively regulated by miR-137-3p and miR-296-5p. (**A**) HT29 cells were transfected with mimics-NC or mimics-miR-137-3p, miR-137-3p expression was determined by qRT-PCR. (**B**) Luciferase activity of wild-type or mutated reporter vector in different groups (NC, mimics-NC, mimics-miR-137-3p). (**C**) SERPINA3 expression was determined in different groups (mimics-NC, mimics-miR-137-3p). (**D**) HT29 cells were transfected with mimics-NC or mimics-miR-296-5p, miR-296-5p expression was determined by qRT-PCR. (**E**) Luciferase activity of wild-type or mutated reporter vector in different groups (NC, mimics-NC, mimics-miR-296-5p). (**F**) SERPINA3 expression was determined in different groups (mimics-NC, mimics-miR-296-5p). N = 3. “*P < 0.05, **P < 0.01 and ***P < 0.001”

To further elucidate the mechanistic actions of SEPRINA3, we further used online tool to predict miRNAs that can potentially target SERPINA3. We found that SERPINA3 3’UTR could bind to miR-137-3p and miR-296-5p (Supplementary Figure S6). Luciferase activity assay showed that miR-137-3p overexpression repressed luciferase activity of wide type vector containing SERPINA3 3’UTR, the mutant one was not affected in HT29 cells (Fig. 8A and B). MiR-137-3p overexpression also down-regulated SERPINA3 expression in HT29 cells (Fig. 8C). Consistently, miR-296-5p overexpression repressed luciferase activity of wide type vector containing SERPINA3 3’UTR, the mutant one was not affected (Fig. 8D and E). Additionally, miR-296-5p overexpression down-regulated SERPINA3 expression in HT29 cells (Fig. 8F).

### Interactive mechanism of miR-137-3p/miR-296-5p and SERPINA3 in the colon cancer cell migration, proliferation and EMT

**Fig. 9:**
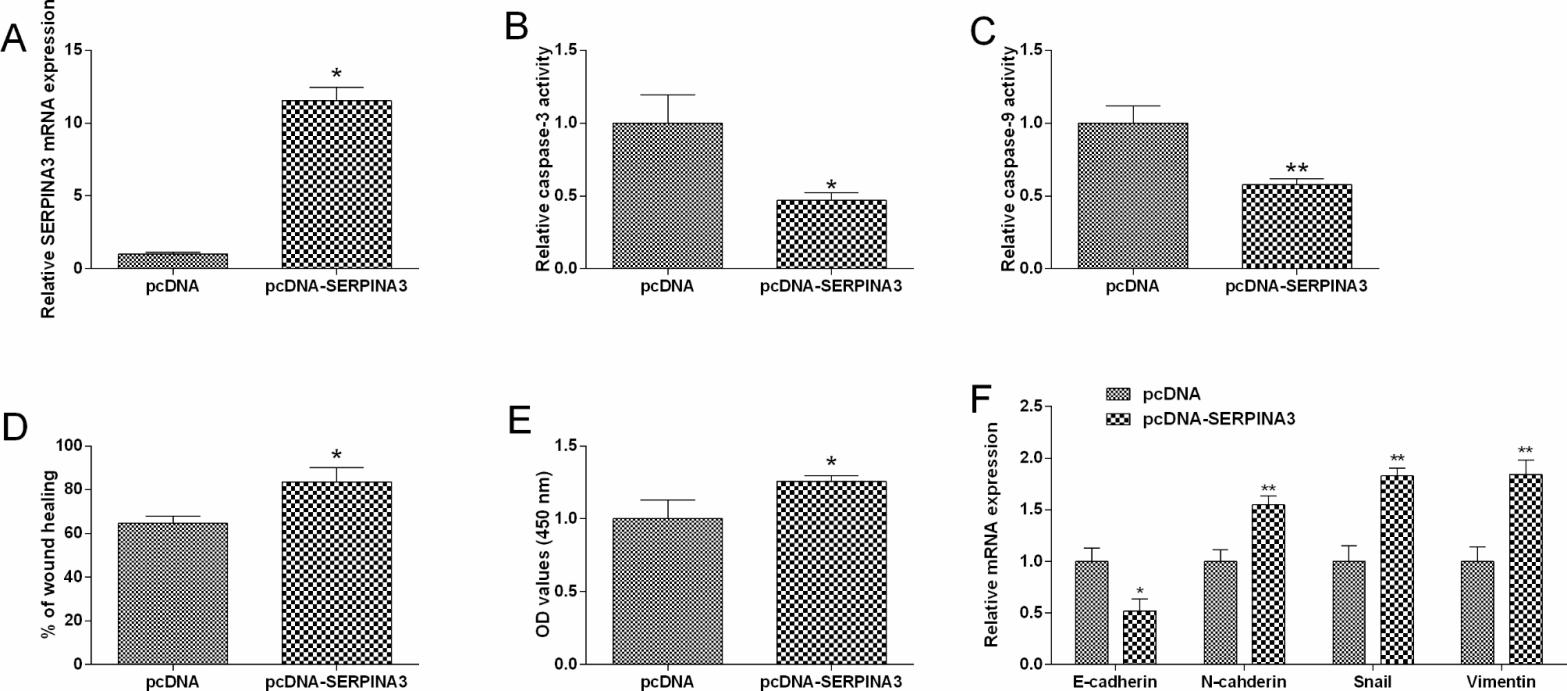
Effects of SERPINA3 overexpression on the colon cancer cell migration, proliferation and EMT. (**A**) HT29 cells were transfected with pcDNA overexpressing SERPINA3 or pcDNA, SERPINA3 expression was determined by evaluated by qRT-PCR. (**B**) HT29 cell caspase-3 activity was determined in different groups (pcDNA, pcDNA-SERPINA3). (**C**) HT29 cell caspase-9 activity was determined in different groups (pcDNA, pcDNA-SERPINA3). (**D**) HT29 cell migratory capacity was determined in different groups (pcDNA, pcDNA-SERPINA3). (**E**) HT29 cell proliferation was determined in different groups (pcDNA, pcDNA-SERPINA3). (**F**) HT29 cell EMT-related marker expression was evaluated in different groups (pcDNA, pcDNA-SERPINA3). N = 3. “*P < 0.05 and **P < 0.01”

Firstly, pcDNA-SEPRINA3 transfection up-regulated SERPINA3 expression in HT29 cells compared to pcDNA group (Fig. 9A). SEPRINA3 overexpression decreased activities of caspase-3/-9, but increased wound healing, proliferation in HT29 cells (Fig. 9B and E). Additionally, pcDNA-SEPRINA3 transfection down-regulated E-cadherin expression, up-regulated N-cadherin, Snail and vimentin expression in HT29 cells (Fig. 9F).

**Fig. 10:**
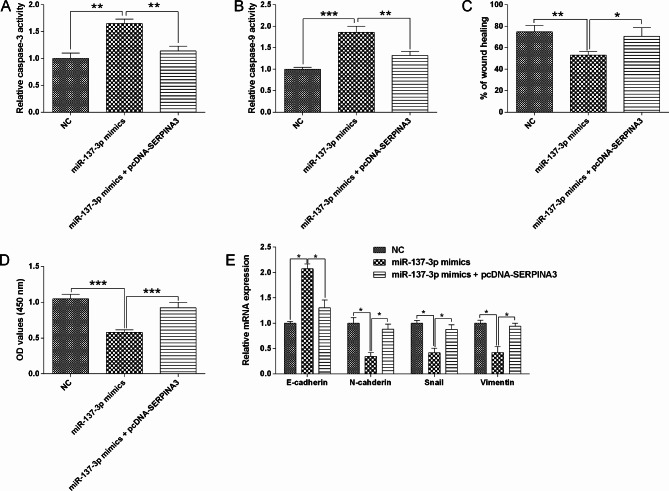
Interactive mechanism of miR-137-3p and SERPINA3 in the colon cancer cell migration, proliferation and EMT. (**A**) HT29 cell caspase-3 activity was determined in different groups. (**B**) HT29 cell caspase-9 activity was determined in different groups. (**C**) HT29 cell migratory capacity was determined in different groups. (**D**) HT29 cell proliferation was determined in different groups. (**E**) HT29 cell EMT-related marker expression was evaluated in different groups (NC, miR-137-3p mimics, miR-137-3p mimics + pcDNA-SERPINA3). N = 3. “*P < 0.05, **P < 0.01 and ***P < 0.001”

**Fig. 11:**
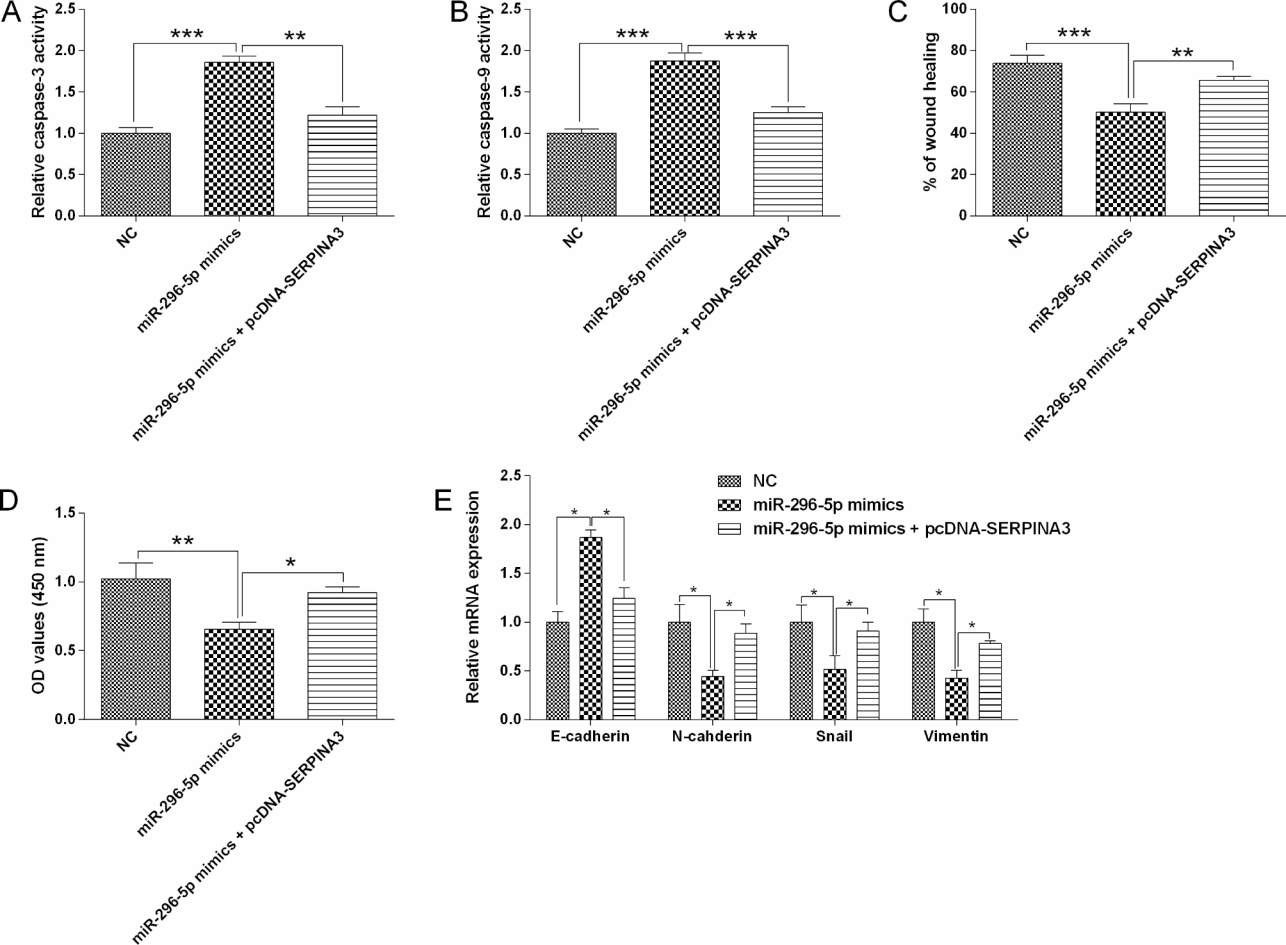
Interactive mechanism of miR-296-5p and SERPINA3 in the colon cancer cell migration, proliferation and EMT. (**A**) HT29 cell caspase-3 activity was determined in different groups. (**B**) HT29 cell caspase-9 activity was determined in different groups. (**C**) HT29 cell migratory capacity was determined in different groups. (**D**) HT29 cell proliferation was determined in different groups. (**E**) HT29 cell EMT-related marker expression was evaluated in different groups. N = 3. “*P < 0.05, **P < 0.01 and ***P < 0.001”

The rescue experiments were further conducted. MiR-137-3p overexpression increased activities of caspase-3/-9, decrease migration and proliferation, and also repressed EMT in HT29 cells, which were obviously attenuated by SERPINA3 enforced overexpression (Fig. 10). Consistently, SERPINA3 enforced overexpression also largely reversed miR-296-5p mimics-induced increase in activities of caspase-3/-9, decrease in migration, proliferation and EMT in HT29 cells (Fig. 11).

## Discussion

Colorectal cancer CRC is a prevalent and deadly malignancy worldwide, with its incidence and mortality projected to rank third among all malignant tumors by 2023 [31]. While advancements in treatment methods have led to improved survival rates and quality of life for CRC patients, the disease continues to be a significant health burden due to its increasing incidence and high mortality rate [32, 33]. Furthermore, the disease is now characterized by a lower age of onset, lower staging, and higher malignancy [3]. Therefore, there is an urgent need to identify the specific molecular mechanisms underlying colorectal cancer metastasis in order to develop effective treatment strategies. This study performed bioinformatics analysis to identify potential genes associated with CRC metastasis. We downloaded and integrated gene expression datasets (GSE89393, GSE100243, and GSE144259) from GEO. Differential expression analysis was conducted, followed by GO analysis and KEGG analysis. The hub gene SERPINA3 was selected for further in vitro functional studies. Additionally, the role of miR-137-3p/miR-296-5p/SERPINA3 in CRC cell function was investigated using in vitro assays. Analysis of the gene expression datasets revealed differentially expressed genes (DEGs) associated with CRC metastasis. GO analysis showed enrichment in biological processes such as blood coagulation regulation and wound healing. Cellular component analysis highlighted extracellular matrix components and secretory granules. Molecular function analysis identified activities such as serine-type endopeptidase inhibition and lipoprotein receptor binding. KEGG analysis revealed involvement in pathways related to complement and coagulation cascades, cholesterol metabolism, and immune responses. The common DEGs among the datasets were further investigated. We identified SERPINA3 as a hub gene associated with CRC metastasis. In vitro assays confirmed the role of miR-137-3p/miR-296-5p/SERPINA3 in CRC cell function.

Bioinformatics has emerged as a valuable interdisciplinary field for acquiring, processing, analyzing, and interpreting biological information using computational methods. In recent years, bioinformatics analysis has played a crucial role in identifying genes associated with tumor occurrence and development. Researchers have utilized various database analysis methods to identify potential tumor markers and shed light on the underlying mechanisms of colorectal cancer metastasis. Regarding GSE89393, Goryca et al. conducted a comprehensive transcriptome sequencing of seven liver metastases, along with their corresponding primary tumors and normal tissue from CRC patients. Their study provided a detailed map of genetic alterations at the exome level that promote metastasis in CRC [34]. As for GSE100243, research studies focused on characterizing the transcriptome of locally advanced colon cancer on a genomic scale. Paraffin-embedded samples were utilized to compare normal colon tissue, primary colon tumors, and lymph node metastases in GSE100243. In the case of GSE144259, the study involved isolating RNA from primary colon cancer, liver metastatic colon cancer, and matched normal colon tissues obtained from the same patient. Through RNA-seq analyses, it was discovered that primary tumors release extracellular vesicles enriched with integrin subunit beta like 1, which facilitates the growth of distal metastatic tumors by creating a fibroblast-niche environment [35]. We explored potential genes associated with CRC metastasis using bioinformatics analysis. We collected gene expression data from CRC patients using publicly available datasets, namely GSE89393, GSE100243, and GSE144259. Through comprehensive data processing and integration, we identified DEGs between cancerous and metastatic tissues. Subsequently, we performed GO analysis and KEGG analysis to gain insights into potential functions of these DEGs. GO analysis revealed enrichment in various biological processes, cellular components, and molecular functions related to tumor progression and metastasis. Additionally, the KEGG analysis identified key pathways involved in CRC metastasis, such as complement and coagulation cascades, platelet activation, and cholesterol metabolism.

In order to gain a better understanding into a specific gene in CRC metastasis, we conducted in vitro functional studies focusing on SERPINA3. The involvement of SERPINA3 has been implicated in various types of cancer. Zhang et al. demonstrated that SERPINA3 promotes tumor progression and EMT in breast cancer cells [36]. Yuan et al. revealed that down-regulated SERPINA3 indicates better prognosis and is associated with immune modulation in glioma [37]. Li et al. found that SERPINA3 could facilitate glioblastoma stem-like cell invasion [38]. Long et al. conducted a meta-analysis of in vitro studies and identified SERPINA3 as a potential novel biomarker candidate for CRC metastasis [39]. In our study, we consistently observed that SERPINA3 exhibited enhanced effects on migration, proliferation, and EMT in CRC cells, while inhibiting caspase-3/-9 activities. These findings suggest the oncogenic actions of SERPINA3 in CRC.

To further elucidate the regulatory mechanisms of SERPINA3, we discovered that miR-137-3p and miR-286-5p both target SERPINA3 3’UTR and repress its expression. Studies have demonstrated that miR-137-3p exerts tumor-suppressive effects in various cancers [40–43]. In CRC, Ding et al. demonstrated that miR-137-3p inhibits CRC cell migration by modulating EMT [44]. As for miR-296-5p, Yan et al. showed that it inhibits cell proliferation by targeting HMGA1 in colorectal cancer [45]. Han et al. revealed that miR-296-5p can inhibit CRC cell metastasis by targeting STAT3 [46]. MiR-137-3p overexpression increased activities of caspase-3/-9, decreased migration and proliferation, and also repressed EMT in HT29 cells, which were obviously attenuated by SERPINA3 enforced overexpression. Consistently, SERPINA3 enforced overexpression also largely reversed miR-296-5p mimics-induced increased in activities of caspase-3/-9, decrease in migration, proliferation and EMT in HT29 cells. Our in vitro assays involving miR-137-3p/miR-296-5p/SERPINA3 revealed valuable insights into the role of this gene in CRC cell function.

## Conclusions

In conclusion, our bioinformatics analysis identified a set of DEGs associated with CRC metastasis, providing valuable insights into the underlying molecular mechanisms. The GO and KEGG enrichment analyses shed light on the biological processes and pathways involved in CRC metastasis, highlighting potential targets for future therapeutic interventions. The functional studies focusing on SERPINA3/miR-137-3p/miR-296-5p further consolidated its role in regulating CRC progression. However, the role of SERPINA3/miR-137-3p/miR-296-5p signaling in CRC still requires further investigation.

### Electronic supplementary material

Below is the link to the electronic supplementary material.

**Supplementary Material 1: Supplementary Figure S1**. The association between PPS of patients with colon cancer and common DEGs. **Supplementary Figure S2**. The association between RFS of patients with colon cancer and common DEGs. **Supplementary Figure S3**. The expression profiles of common DEGs between cancerous and normal groups. **Supplementary Figure S4**. The expression of SERPINA3 in the colon cancer tissues with different clinical stages. **Supplementary Figure S5**. The expression of SERPINA3 in various immune cells by CIERSORT (GEPIA2021). **Supplementary Figure S6**. Prediction for targets between SERPINA3 3’UTR and miR-137-3p/miR-296-5. (A) MiR-137-3p-targeted sequence of SERPINA3 3’UTR. (B) MiR-296-5p-targeted sequence of SERPINA3 3’UTR

## Data Availability

All the data are available upon reasonable request from corresponding author. GSE89393 (https://www.ncbi.nlm.nih.gov/geo/query/acc.cgi?acc=GSE89393_, GSE100243 (https://www.ncbi.nlm.nih.gov/geo/query/acc.cgi?acc=GSE100243) and GSE144259 (https://www.ncbi.nlm.nih.gov/geo/query/acc.cgi?acc=GSE144259).
