# Supplementary material for: Unveiling the role of miR-137-3p/miR-296-5p/SERPINA3 signaling in colorectal cancer progression: integrative analysis of gene expression profiles and in vitro studies
Source: BMC Med Genomics. 2023 Dec 12;16:327. doi: 10.1186/s12920-023-01763-w (PMC10714458; doi:10.1186/s12920-023-01763-w)
Supplement: Supplementary file 1 — Supplementary Material 1: Supplementary Figure S1. The association between PPS of patients with colon cancer and common DEGs. Supplementary Figure S2. The association between RFS of patients with colon cancer and common DEGs. Supplementary Figure S3. The expression profiles of common DEGs between cancerous and normal groups. Supplementary Figure S4. The expression of SERPINA3 in the colon cancer tissues with different clinical stages. Supplementary Figure S5. The expression of SERPINA3 in various immune cells by CIERSORT (GEPIA2021). Supplementary Figure S6. Prediction for targets between SERPINA3 3’UTR and miR-137-3p/miR-296-5. (A) MiR-137-3p-targeted sequence of SERPINA3 3’UTR. (B) MiR-296-5p-targeted sequence of SERPINA3 3’UTR [file 12920_2023_1763_MOESM1_ESM.pdf]

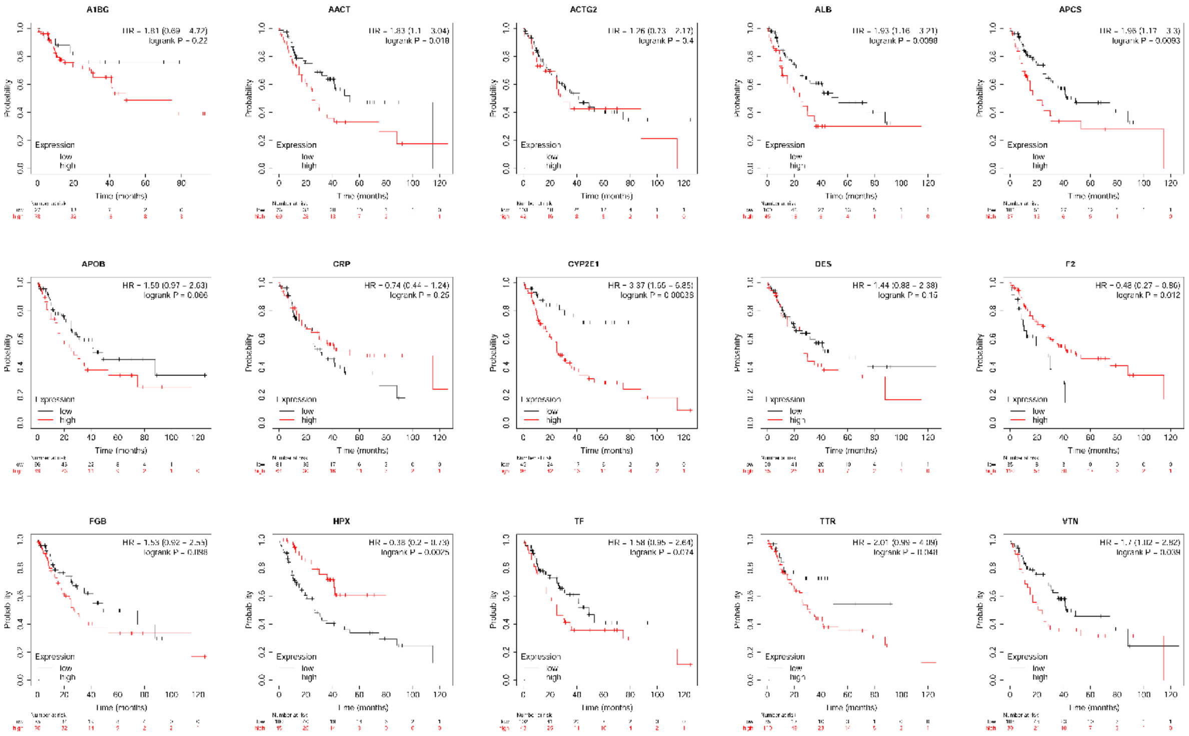

Supplementary Figure S1. The association between PPS of patients with colon cancer and common DEGs.

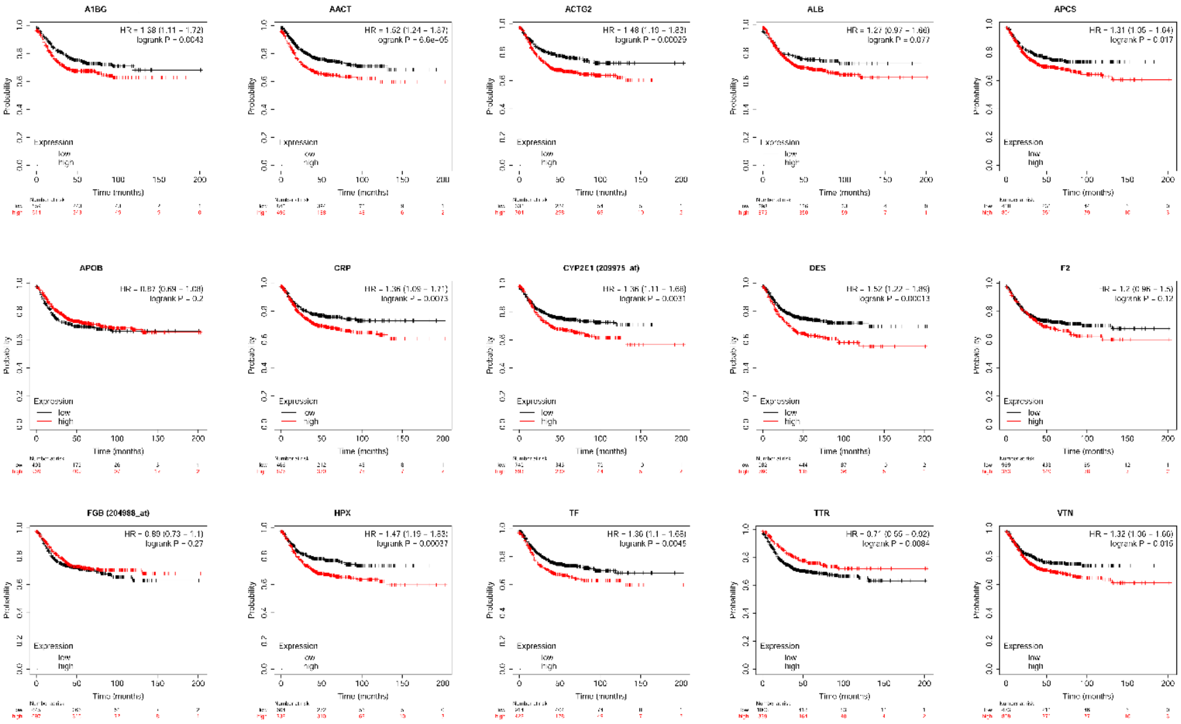

Supplementary Figure S2. The association between RFS of patients with colon cancer and common DEGs.

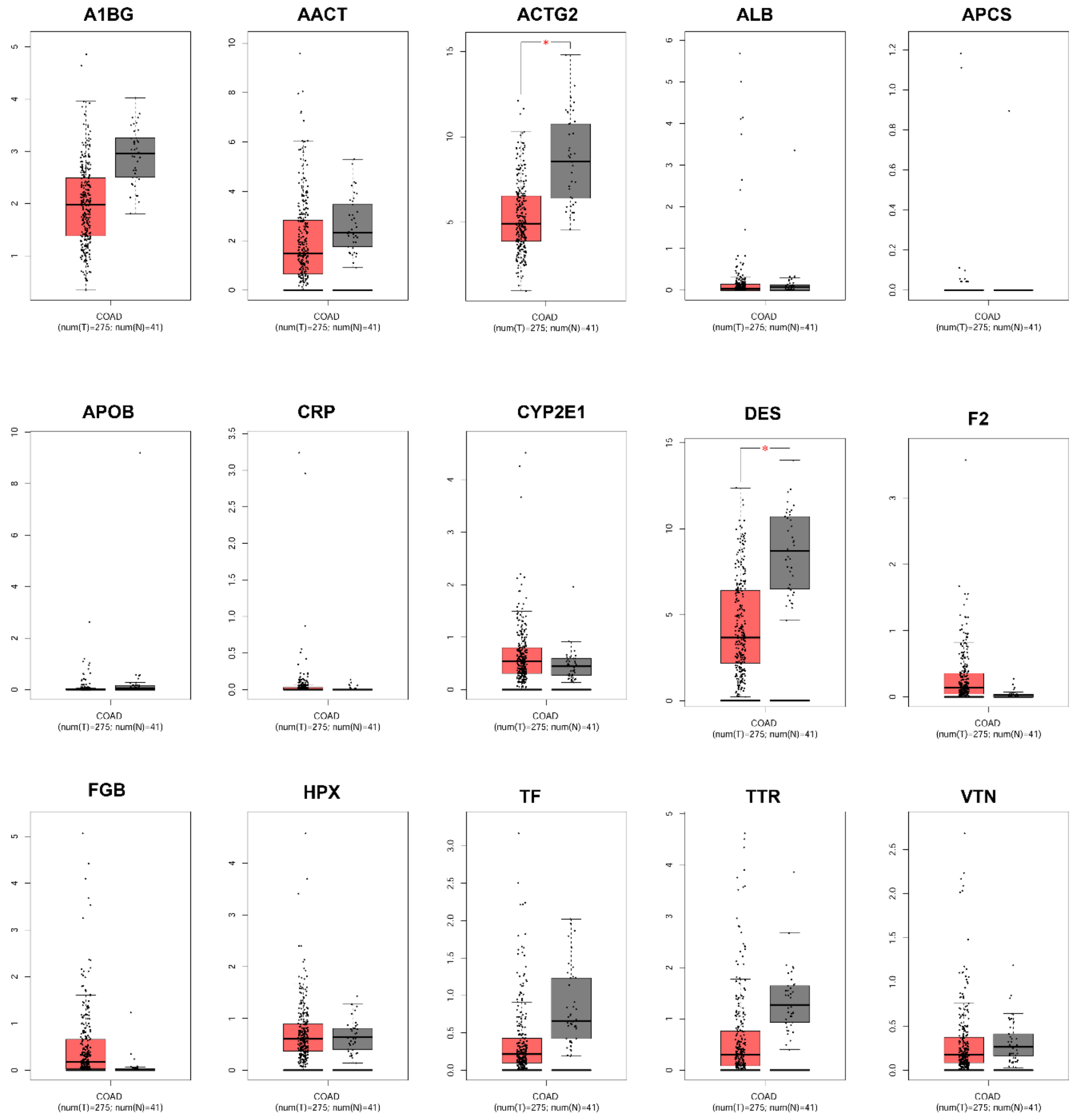

Supplementary Figure S3. The expression profiles of common DEGs between cancerous and normal groups.

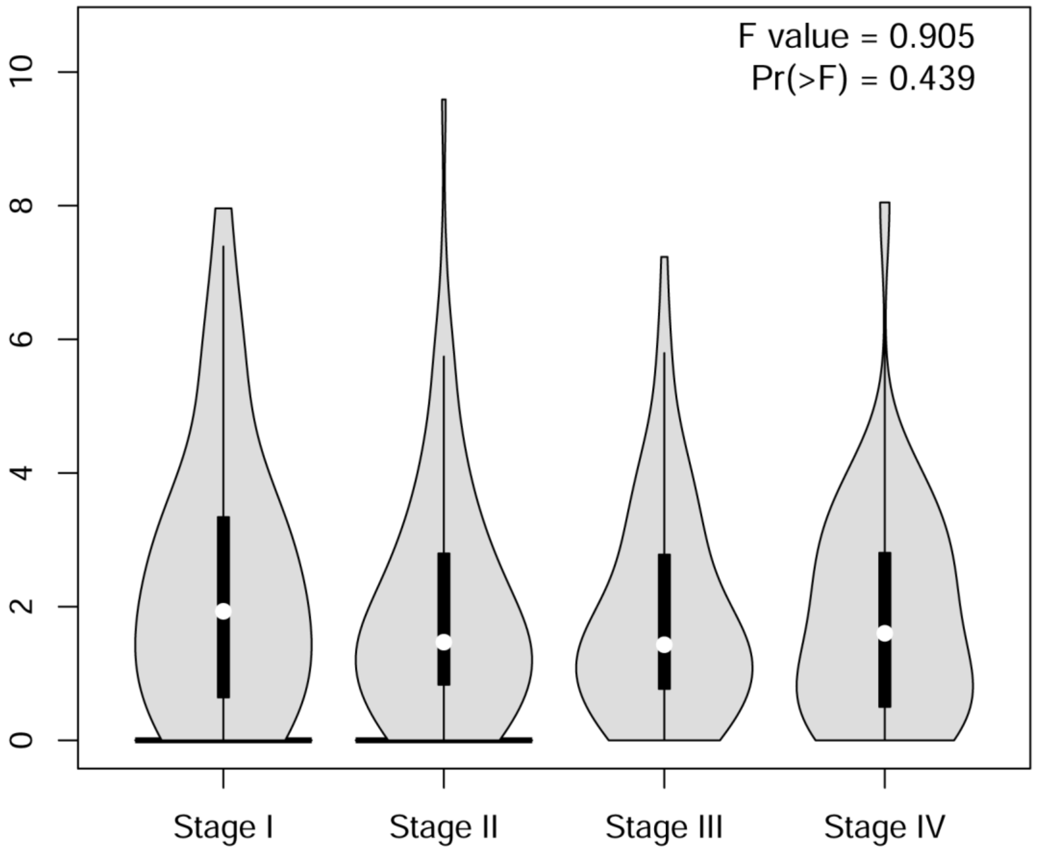

Supplementary Figure S4. The expression of SERPINA3 in the colon cancer tissues with different clinical stages.

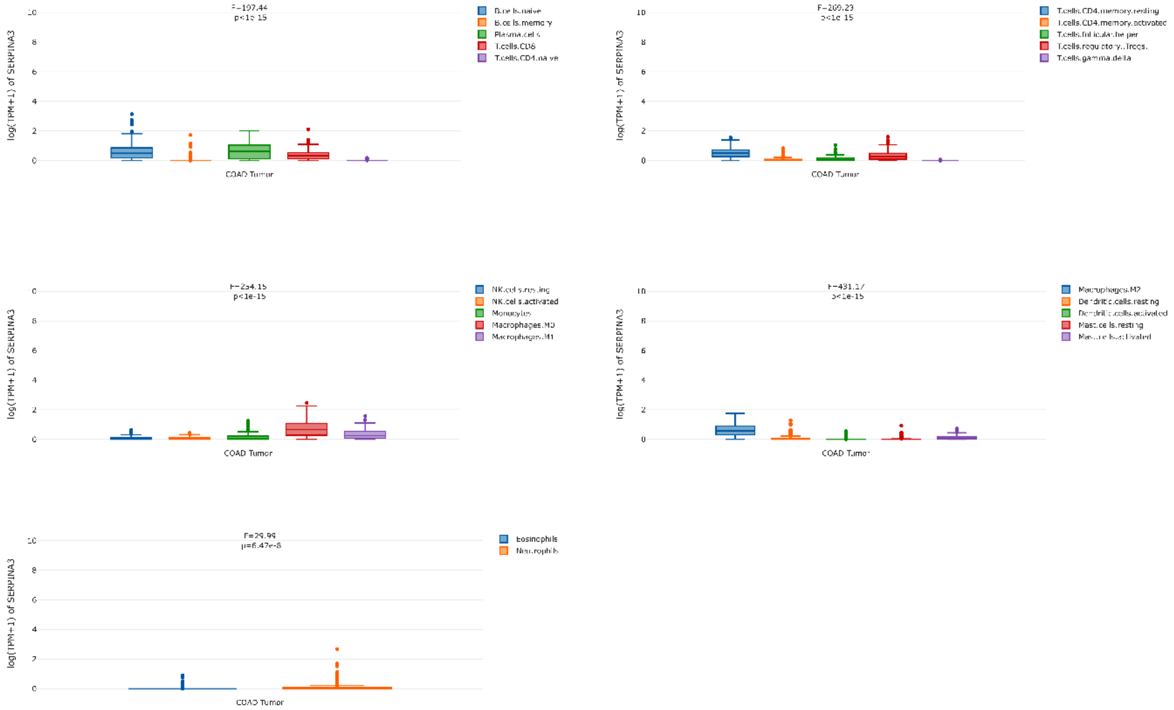

Supplementary Figure S5. The expression of SERPINA3 in various immune cells by CIERSORT (GEPIA2021).

A	SERPINA3 3'UTR (WT)	3'	AAUAACGACAGUC........	5'
	miR-137-3p	5'	UUAUUGCUUAAGAAUACGCGUAG	3'
	SERPINA3 3'UTR (Mut)	3'	AAUUUGGACAGUC........	5'
B	SERPINA3 3'UTR (WT)	3'	ACCCGGGGACAC........	5'
	miR-296-5p	5'	AGGGCCCCCCCUCAAUCCUGU	3'
	SERPINA3 3'UTR (Mut)	3'	ACCGCCGGACAC........	5'

Figure S6. Prediction for targets between SERPINA3 3'UTR and miR-137-3p/miR-296-5. (A) MiR-137-3p-targeted sequence of SERPINA3 3'UTR. (B) MiR-296-5p-targeted sequence of SERPINA3 3'UTR.
